# Krüppel-like factor 4 regulates genetic stability in mouse embryonic fibroblasts

**DOI:** 10.1186/1476-4598-12-89

**Published:** 2013-08-06

**Authors:** Enas A El-Karim, Engda G Hagos, Amr M Ghaleb, Bing Yu, Vincent W Yang

**Affiliations:** 1 Department of Medicine, HSC T-16, Rm 020, Stony Brook University, Stony Brook, NY 11794, USA; 2 Department of Biology, Colgate University, Hamilton, NY 13346, USA

**Keywords:** KLF4, Genetic instability, DNA damage responses, Aneuploidy, Centrosome amplification, Mouse embryonic fibroblasts

## Abstract

**Background:**

Krüppel-like factor 4 (KLF4) is a member of the KLF family of transcription factors and regulates proliferation, differentiation, apoptosis and somatic cell reprogramming. Evidence also suggests that KLF4 is a tumor suppressor in certain cancers including colorectal cancer. We previously showed that KLF4 inhibits cell cycle progression following DNA damage and that mouse embryonic fibroblasts (MEFs) null for *Klf4* are genetically unstable, as evidenced by increased rates of cell proliferation, and the presence of DNA double strand breaks (DSBs), centrosome amplification, chromosome aberrations and aneuploidy.

**Methods:**

To determine whether re-expression of Klf4 corrects the observed genetic instability in MEFs null for *Klf4* (*Klf4*^*−/−*^), we transfected *Klf4*^*−/−*^MEFs with Klf4-expressing plasmids and compared the results to wild type (*Klf4*^*+/+*^) and untransfected or mock-transfected *Klf4*^*−/−*^MEFs.

**Results:**

We show that overexpression of Klf4 in *Klf4*^*−/−*^MEFs reduced cell proliferation rates and the proportion of cells with DSBs, abnormal centrosome numbers, aneuploidy and micronuclei. In addition, Klf4-transfected *Klf4*^*−/−*^MEFs exhibited a more robust DNA damage repair response as demonstrated by the greater rate in disappearance of γ-H2AX and 53BP1 foci following γ-irradiation.

**Conclusion:**

Taken together these findings provide evidence that KLF4 plays a crucial role in the maintenance of genetic stability by modulating the DNA damage response and repair processes.

## Background

Krüppel-like factor 4 (KLF4) [1,2] belongs to the Krüppel-like factor family of zinc-finger transcription factors that are involved in numerous important cellular processes such as growth, development, differentiation, proliferation, inflammation, apoptosis, and somatic cell reprogramming. KLF4 has been shown in a context-dependent manner to be an oncogene or tumor suppressor [3], as respectively demonstrated by the high levels of KLF4 in primary breast ductal carcinoma and oral squamous cell carcinoma [4,5] and decreased levels of KLF4 in a variety of other human cancers including esophageal, gastric, bladder, pancreatic, colorectal, lung and urinary bladder cancers [6-14].

We and others have reported that one of the functions of KLF4 is to maintain the proper progression and integrity of the cell cycle [15]. KLF4 inhibits cell proliferation by functioning as a cell cycle checkpoint protein to activate transcription of the cyclin-dependent kinase inhibitor, p21 [1,16]. Additionally, KLF4 is an important mediator of p53-dependent growth arrest in the G_1_/S and G_2_/M transitions of the cell cycle following DNA damage [17]. More recently, we reported that KLF4 is important for the maintenance of genetic stability. This was demonstrated by the appearance of genetic instability in mouse embryonic fibroblasts (MEFs) null for the *Klf4* gene in the forms of increased DNA double strand breaks (DSBs), chromosomal aberrations and centrosome amplification [18]. Since genetic instability plays a crucial role in the development and progression of human cancer [19], we sought to determine whether re-expression of Klf4 in *Klf4*^*−/−*^MEFsMay correct the observed genetic instability in these cells.

## Results

### Re-expression of Klf4 in *Klf4*^*−/−*^MEFs reduces the rate of cell proliferation

**Figure 1:**
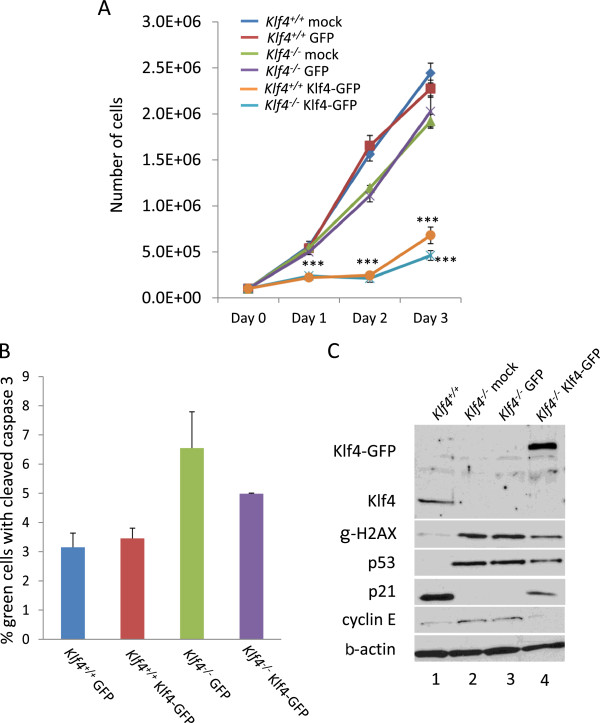
**Growth characteristics of Klf4-transfected *Klf4***^***−/−***^**MEFs in culture. (A)** Cell proliferation rates of *Klf4*^*+/+*^ and *Klf4*^*−/−*^MEFs, transfected or not with various plasmids. Cells were initially seeded at 10^5^ cells/plate in 60 mm plates. On day 0, cells were transfected in triplicates with pmKLF4-pEGFP, GFP control, or mock-transfected in parallel. Number of cells was counted (three plates per transfection) every day over a total of 3 days. The growth rate of the MEFs from each transfection was determined at each time point and the values represent the mean number of cells per well. N = 3 (*** = *p* < 0.001 for *Klf4*^*+/+*^ and *Klf4*^*−/−*^ cells transfected with Klf4-GFP compared to mock- or GFP-transfected *Klf4*^*+/+*^ and *Klf4*^*−/−*^ cells, respectively). **(B)** Quantification of apoptotic *Klf4*^*+/+*^ and *Klf4*^*−/−*^MEFs following GFP or Klf4-GFP overexpression. At 24 h post-transfection, immunostaining for both cleaved caspase 3 and GFP was done and number of cells that are positive for both were counted. N = 3 (Difference did not reach significance for *Klf4*^*−/−*^ cells transfected with Klf4-GFP compared to GFP-transfected *Klf4*^*−/−*^ cells, *p =* 0.059). **(C)** Western blot analysis of Klf4, p53, p21, cyclin E, γH2AX, and β-actin in protein extracts at 24 h post transfection of *Klf4*^*+/+*^ and *Klf4*^*−/−*^ cells with GFP or Klf4-GFP.

MEFs deficient for *Klf4* are known to have a higher rate of BrdU incorporation and apoptosis relative to wild type cells [18]. We assessed whether re-expression of Klf4 in *Klf4*^*−/−*^MEFs will affect the proliferative capacity and or apoptosis. We first compared the growth rates of Klf4-GFP-transfected *Klf4*^*−/−*^ to mock- or GFP-transfected *Klf4*^*−/−*^MEFs and to mock-, GFP-, or Klf4-GFP-transfected *Klf4*^*+/+*^MEFs up to three days after transfection. As shown in Figure 1A, in Klf4-GFP-transfected *Klf4*^*−/−*^MEFs, cell proliferation was significantly reduced compared to mock-transfected or GFP-control-transfected *Klf4*^*−/−*^ or *Klf4*^*+/+*^ cells up to three days post-transfection. Proliferation of Klf4-GFP-transfected *Klf4*^*+/+*^MEFs was also significantly reduced compared to mock-transfected or GFP-control-transfected *Klf4*^*−/−*^ and *Klf4*^*+/+*^ cells. Recently, we demonstrated that *Klf4*^*−/−*^MEFs have a higher level of apoptosis than *Klf4*^*+/+*^MEFs [18]. We examined whether re-expression of Klf4 in *Klf4*^−/−^MEFs has any effect on apoptosis level. We transfected Klf4-GFP or GFP-control in *Klf4*^*+/+*^ and *Klf4*^*−/−*^MEFs, immunostained them for cleaved caspase 3, and counted the number of GFP-positive cells that were positive for cleaved caspase 3. As shown in Figure 1B, overexpression of Klf4 has no apparent effect on the basal level of apoptosis in *Klf4*^*+/+*^MEFs transfected with Klf4-GFP as compared to the control. Although overexpression of Klf4 in *Klf4*^*−/−*^MEFs lowered the apoptosis level compared to GFP-control-transfected *Klf4*^−/−^ cells, it did not reach statistically significant value (*p* = 0.059).

KLF4 has been shown to play an important role in the regulation of cell cycle progression through modification of expression levels of multiple proteins (e.g. p21, p53) [3,20,21]. Since re-expressing Klf4 in *Klf4*^*−/−*^MEFs reduced proliferation (Figure 1A), we hypothesized that re-expression of Klf4 in *Klf4*^*−/−*^MEFs will affect the levels of proteins involved in cell proliferation including p21. To validate this hypothesis, we performed Western blot analysis in *Klf4*^*+/+*^, mock- or GFP-control-transfected *Klf4*^*−/−*^ and Klf4-GFP-transfected *Klf4*^*−/−*^MEFs’s. Additionally we analyzed the levels of proteins involved in centrosome amplification (p53 and cyclin E) and DNA damage (γ-H2AX). It was previously shown that *Klf4*^*−/−*^*M*EFs have lower levels of p21, increased levels of p53, cyclin E, and γ-H2AX proteins in comparison to *Klf4*^*+/+*^MEFs [18,22,23]. As shown in Figure 1C, relative to *Klf4*^+/+^ cells, Western blot analysis of mock- or GFP-control-transfected *Klf4*^*−/−*^MEFs showed an absence of p21, and an increase in p53, cyclin E, and γ-H2AX levels, similar to previous studies [18,22,23]. In contrast, Klf4-GFP-transfected *Klf4*^−/−^ cells had a higher level of p21 and reduced levels of p53, cyclin E and γ-H2AX when compared to mock- or GFP-control-transfected *Klf4*^−/−^ cells (Figure 1C). These results indicate that re-expression of Klf4 in *Klf4*^−/−^MEFs trends toward restoring the levels of cell cycle regulatory proteins and γ-H2AX to that of wild-type cells.

### Re-expression of Klf4 in *Klf4*^*−/−*^MEFs corrects centrosome amplification

**Figure 2:**
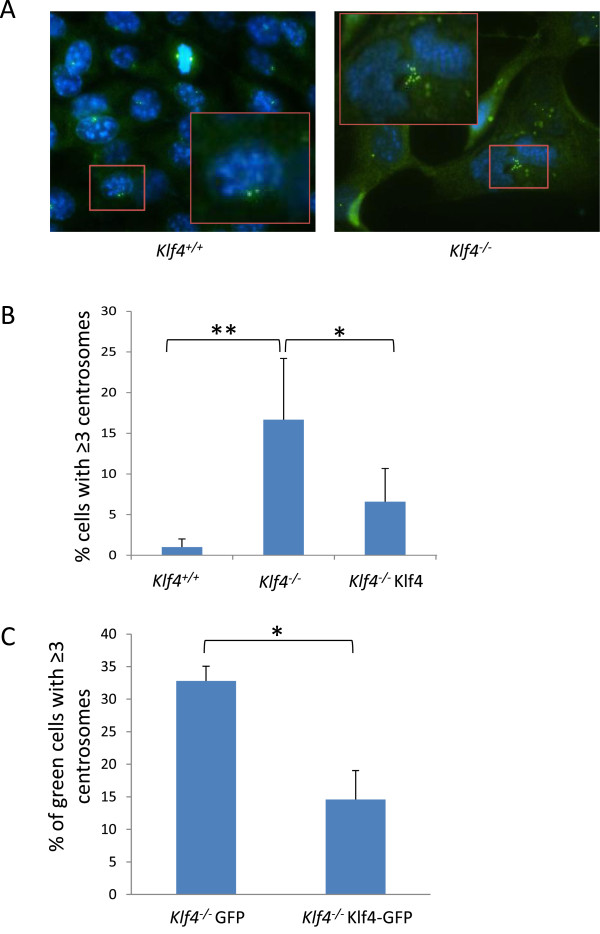
**Overexpression of Klf4 suppresses centrosome amplification in *Klf4***^**−/−**^**MEFs.** Centrosome staining was conducted with an antibody against γ-tubulin and detected with Alexa Fluor 488-conjugated antibody. Hoechst stain (blue) was used to visualize the nuclei. **(A)** A representative image of centrosome staining of *Klf4*^*+/+*^ and *Klf4*^*−/−*^ MEFs. The inset shows a *Klf4*^*+/+*^ cell with 2 centrosomes and a *Klf4*^*−/−*^ cell with abnormal number of centrosomes (≥ 3). Shown is a typical result of 4 independent experiments. **(B)** histogram showing quantification in percentages of cells with ≥3 centrosomes in *Klf4*^*+/+*^*, Klf4*^*−/−*^*,* and GFP or Klf4-GFP transfected *Klf4*^*-/ -*^MEFs. One hundred cells were counted per cell type per experiments. N = 5; * *p* < 0.05, ** *p* < 0.01 compared to *Klf4*^*+/+*^ cells. **(C)** A graph showing the percentage of green *Klf4*^*−/−*^ cells (positive for GFP-control or Klf4-GFP) with ≥3 centrosomes. One hundred green cells were counted per cell type per experiments. N = 5; * *p* < 0.05 compared to *Klf4*^*−/−*^ cells transfected with GFP alone.

We previously showed that Klf4 plays a role in regulating centrosome duplication in MEFs - whereas 2-3% of *Klf4*^+/+^ MEFs exhibited centrosome amplification, defined as the presence of 3 or more centrosomes per cell, approximately 25% of the *Klf4*^*−/−*^MEFs had centrosome amplification, [18]. To determine if re-expressing Klf4 corrects the numerical centrosome abnormality in *Klf4*^*−/−*^MEFs, we performed immunofluorescent staining of centrosomes with an antibody against γ-tubulin. An example of the results of such staining in *Klf4*^*+/+*^ and *Klf4*^*−/−*^ is shown in Figure 2A, which demonstrates the normal distribution of 2 centrosomes per cell in *Klf4*^*+/+*^MEFs but up to 8 centrosomes in a *Klf4*^*−/−*^MEF. We quantified the total number of cells with 3 or more centrosomes in *Klf4*^*+/+*^*,* GFP-control-transfected *Klf4*^*−/−*^*,* and Klf4-GFP-transfected *Klf4*^*−/−*^MEFs. As shown in Figure 2B*,* there was a significant increase in cells with 3 or more centrosomes in GFP-transfected *Klf4*^*−/−*^ as compared to *Klf4*^*+/+*^ cells (2-3% and 17%, respectively). Overexpression of Klf4 in *Klf4*^*−/−*^MEFs significantly reduced the number of cells with 3 or more centrosomes to an average of 7%. To further demonstrate a direct link between Klf4 levels and the extent of centrosome number correction, we overexpressed Klf4-GFP in *Klf4*^*−/−*^MEFs and counted only the cells that were positive for GFP and have ≥3 centrosomes. As shown in Figure 2C, overexpression of Klf4-GFP in *Klf4*^*−/−*^MEFs resulted in a significant decrease in the percentage of cells with ≥3 centrosomes compared to GFP-control-transfected *Klf4*^*−/−*^MEFs (12% and 32%, respectively).

### Klf4 re-expression reduces γ-irradiation-induced DNA damage in *Klf4*^-/-^ MEFs

**Figure 3:**
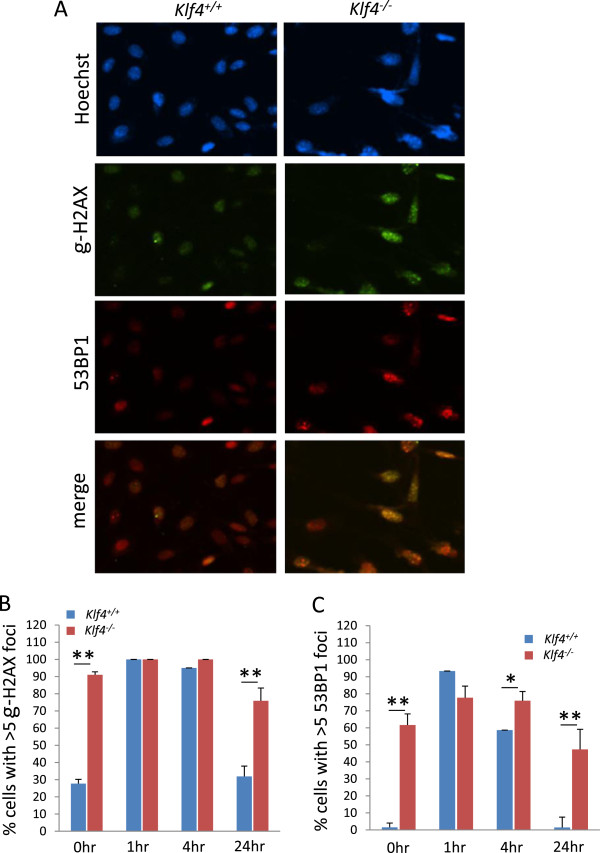
**Immunostaining for γ-H2AX and 53BP1 in *Klf4***^***+/+ ***^**and *Klf4***^***−/−***^**MEFs. (A)** Immunostaining was conducted for γ-H2AX and 53BP1 in *Klf4*^*+/+*^ and *Klf4*^*−/−*^MEFs. Non-irradiated *Klf4*^*+/+*^ and *Klf4*^*−/−*^MEFs were stained with antibodies against γ-H2AX and 53BP1 and hoechst stain (blue) was used to visualize nuclei. Shown is a representative result of three independent experiments. **(B)** Histogram showing quantification of cells with ≥ 5 γ-H2AX foci in *Klf4*^*+/+*^ and untransfected *Klf4*^*−/−*^MEFs with and without γ-irradiation. **(C )** Histogram showing quantification of cells with ≥ 5 53BP1 foci in *Klf4*^*+/+*^ and untransfected *Klf4*^*−/−*^MEFs with and without γ-irradiation. For **(B)** and **(C)**, foci were counted for non-irradiated cells, and at 1, 4 and 24h post irradiation. One hundred cells were counted per cell type per experiment. N = 5; * *p* < 0.05, ** *p* < 0.01 compared to *Klf4*^*+/+*^ cells.

To determine the role of Klf4 in the DNA damage response and repair process, we first evaluated the extent of double strand breaks (DSBs) with and without γ-irradiation in *Klf4*^*+/+*^ and *Klf4*^*−/−*^*M*EFs using γ-H2AX and 53BP1 as markers of DNA damage. We induced DNA damage with γ-irradiation and counted cells with 5 or more foci for each marker at 0, 1, 4, and 24h post γ-irradiation. An example of γ-H2AX and 53BP1 staining in non-irradiated *Klf4*^*+/+*^ and *Klf4*^*−/−*^MEFs is shown in Figure 3A. Approximately 25% of non-irradiated *Klf4*^*+/+*^MEFs and 90% of *Klf4*^*−/−*^MEFs had ≥5 γ-H2AX foci (Figure 3B). The number of cells with ≥5 γ-H2AX foci was significantly increased at 1 and 4h post-irradiation in *Klf4*^*+/+*^MEFs and then returned to the basal level by 24 h post-irradiation. In contrast, the number of cells with ≥5 γ-H2AX foci remained elevated in *Klf4*^−/−^MEFs up to 24 h post-irradiation. A similar trend was noted for cells with ≥5 53BP1 foci in the two MEFs before and after irradiation (Figure 3C). These results suggest that while wild-type MEFs exhibit a normal DNA damage response following γ-irradiation, cells lacking *Klf4 *have persistent evidence of DNA damage.

**Figure 4:**
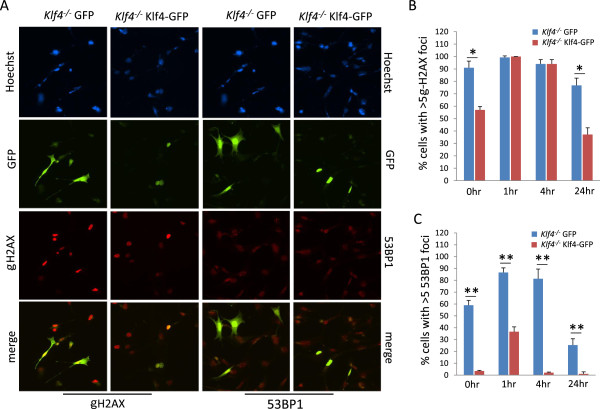
**Immunostaining for γ-H2AX and 53BP1 in *Klf4***^***−/−***^**MEFs transfected with GFP or Klf4-GFP. (A)** Immunostaining was conducted for γ-H2AX and 53BP1 in *Klf4*^*−/−*^MEFs transfected with GFP or Klf4-GFP.hoechst stain (blue) was used to visualize nuclei. Shown is a representative result of three independent experiments for the non-irradiated cells. **(B)** Histogram showing quantification of cells with ≥ 5 γ-H2AX foci in *Klf4*^*−/−*^MEFs cells transfected with GFP or Klf4-GFP, with or without γ-irradiation. **(C)** Histogram showing quantification of cells with ≥ 5 53BP1 foci in *Klf4*^*−/−*^MEFs cells transfected with GFP or Klf4-GFP, with or without γ-irradiation. For **(B)** and **(C)**, foci were counted for non-irradiated cells, and at 1, 4 and 24 h post irradiation. One hundred green cells were counted per cell type per experiment. N = 5; * *p* < 0.05, ** *p* < 0.01 compared to GFP-transfected *Klf4*^*−/−*^ cells.

We then determined if re-expression of Klf4 corrects the DNA damage observed in *Klf4*^*−/−*^MEFs by transfecting GFP-control or Klf4-GFP into *Klf4*^*−/−*^ cells. Figure 4A shows an example of GFP-positive green cells with γ-H2AX and 53BP1 staining in GFP- or Klf4-GFP-transfected *Klf4*^−/−^MEFs. As shown in Figure 4B, at baseline, transfection of *Klf4*^−/−^ cells with Klf4-GFP significantly reduced the number of green cells with γ-H2AX foci as compared to GFP-transfected cells. At 1 and 4 h after irradiation, the number of cells with γ-H2AX foci increased in Klf4-GFP-transfected *Klf4*^−/−^MEFs and then returned to a level below that of GFP-transfected MEFs at 24 h post-irradiation. A similar trend is noted for 53BP1 except that Klf4-GFP-transfectedMEFs had lower percentage of cells with ≥5 53BP1 foci relative to GFP-transfected cells at all time-points (Figure 4C). These data suggest that re-expression of Klf4 in *Klf4*^−/−^MEFs results in a more efficient repair of DNA damage compared to control *Klf4*^−/−^ cells.

### Klf4 re-expression reduces aneuploidy due to *Klf4* deletion

**Figure 5:**
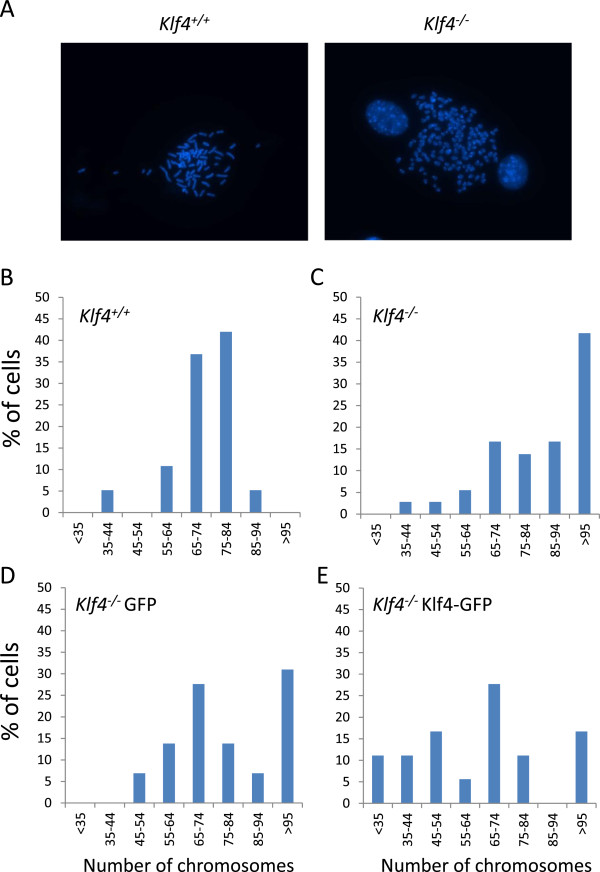
**Determination of ploidy in *Klf4***^**+/+ **^**and *Klf4***^***−/−***^**MEFs.** Karyotype analysis was conducted in metaphase chromosome spreads prepared from *Klf4*^*+/+*^ and from *Klf4*^*−/−M*^EFs transfected or not with GFP or Klf4-GFP. **(A)** Shown is a typical result of three independent experiments for *Klf4*^*+/+*^ and *Klf4*^*−/−*^MEFs. **(B**-**E)** Histograms showing quantification of chromosome number in *Klf4*^*+/+*^ and in *Klf4*^*−/−*^MEFs untransfected or transfected with GFP or Klf4-GFP. Spreads from 100 cells were examined per genotype.

We recently reported that *Klf4*^*−/−*^MEFs exhibited genetic instability manifested by the presence of aneuploidy [18]. To determine whether re-expression of Klf4 corrects this abnormality, we transfected *Klf4*^*−/−*^MEFs with Klf4-expressing plasmids and performed cytogenetic analysis. An example of metaphase chromosome spreads in *Klf4*^*+/+*^ and *Klf4*^*−/−*^ is shown in Figure 5A. As seen in Figure 5B, analysis of metaphase chromosome spreads demonstrated that while *Klf4*^*+/+*^MEFs showed a distribution of chromosome numbers between the 35–44 and 75–84 (approximating haploid and diploid, respectively), *Klf4*^*−/−*^MEFs consistently had higher numbers of chromosomes with many cells displaying greater than 95 chromosomes per cell, indicating the presence of aneuploidy. Overexpression of Klf4 in *Klf4*^*−/−*^MEFs, however, resulted in a reduction in the number of cells exhibiting aneuploidy as compared to untransfected *Klf4*^*−/−*^ or GFP-control-transfected *Klf4*^*−/−*^MEFs.

### Klf4 re-expression in *Klf4*-null MEFs decreases the number of micronuclei

**Figure 6:**
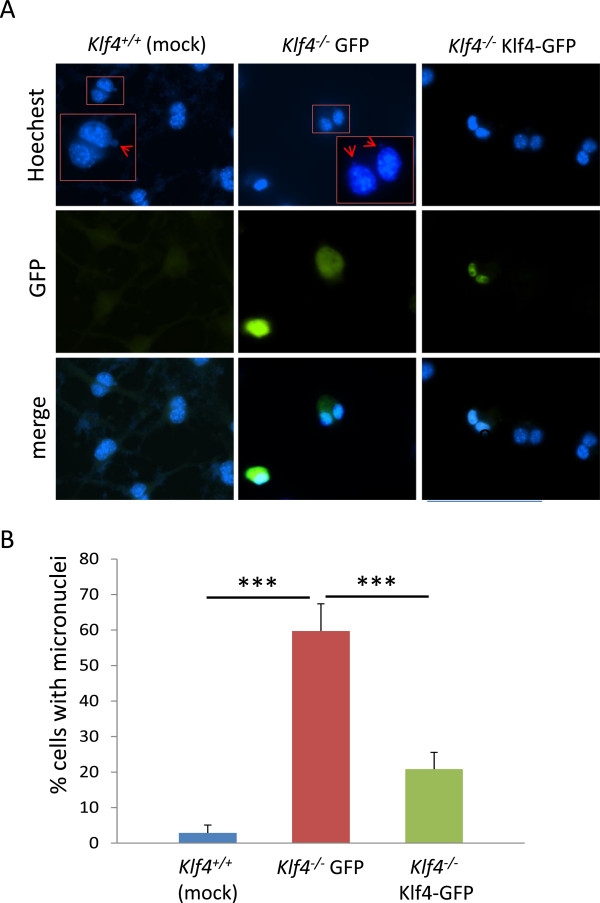
**Determination of micronuclei (MN) formation in MEFs.** Micronuclei (MN) analysis was conducted in cytokinesis-arrested cells prepared from *Klf4*^*+/+*^ and from *Klf4*^*−/−M*^EFs transfected with GFP or Klf4-GFP. **(A)***Klf4*^*+/+*^ and *Klf4*^*−/−*^MEFs transfected with GFP or Klf4-GFP were treated with cytochalasin-B (CCB) and Hoechst stain (blue) was used to visualize nuclei. Shown are representative images of cells containing MN. **(B)** Histogram showing quantification of percent of binucleated cells with MN 24h post CCB treatment. At least 200 green cells were counted for each genotype per experiment. N = 3; *** *p* < 0.001 compared to GFP-transfected *Klf4*^*−/−*^ cells.

Micronuclei are formed due to DSBs and represent a mechanism by which errors in chromosome segregation and DNA breaks are eliminated from the nucleus of the cell [24]. Given the increased proliferation rate, DNA damage and aneuploidy observed in *Klf4*^*−/−*^ in comparison with *Klf4*^*+/+*^MEFs, we further assessed the frequency of micronuclei formation which can originate from DNA breaks, chromosome fragments or lagging chromosomes during aberrant cell division in *Klf4*^*−/−*^MEFs. We used cytochalasin-B, an inhibitor of cytokinesis that allows easily distinguishing between mononucleated non-dividing cells and binucleated dividing cells. To examine the effect of Klf4 on the frequency of binucleated cells containing micronuclei in *Klf4*^*−/−*^MEFs, we overexpressed GFP-control or Klf4-GFP in *Klf4*^*−/−*^MEFs and treated cells with cytochalasin-B. At 24 h following cytochalasin-B addition, we stained cells with Hoechst and analyzed only the green, binucleated cells and quantified the frequency of cells with micronuclei. An example of binucleated cells containing micronuclei staining in *Klf4*^*+/+*^ and binucleated green *Klf4*^*−/−*^ cells is shown in Figure 6A. As shown in Figure 6B, approximately 4% of *Klf4*^*+/+*^ cells have binucleated cells with micronuclei, while 60-70% of GFP-control-transfected *Klf4*^*−/−*^MEFs have binucleated cells with micronuclei. Importantly, re-expression of Klf4-GFP in *Klf4*^*−/−*^MEFs reduced the levels of binucleated cells with micronuclei to 20-30% (Figure 6B).

## Discussion

Previously, we demonstrated that deletion of Klf4 from MEFs cells leads to increased genetic instability. *Klf4*^*−/−*^MEFs display significantly higher levels of DNA damage as indicated by the increased presence of γ-H2AX foci compared with *Klf4*^*+/+*^MEFs with and without γ-irradiation [18,23]. Additionally, the loss of Klf4 leads to defective cell-cycle checkpoint functions, aberrant centrosome duplication and increased aneuploidy. In view of these findings, we set to determine if reintroduction of Klf4 into *Klf4*^*−/−*^MEFs will correct the observed genetic instable phenotype.

Cancer cells generally contain the full complement of biomolecules that are necessary for survival, proliferation, differentiation, and cell death [25]. However, it is the failure to regulate these functions that results in an altered phenotype in cancer. Defects in checkpoint control increase genetic instability and contribute to uncontrolled proliferation. We have previously shown that relative to wild-type cells, MEFs deficient in Klf4 had a higher rate of BrdU incorporation that, was seemingly offset by a higher level of apoptosis [18]. KLF4 is required for cell cycle arrest in G_1_, G_2_ or both in many cell types by modulating expression of cell cycle regulatory genes [16,17,26]. Moreover, transcriptional profiling of KLF4 in cell lines suggests that KLF4 functions as a negative regulator of cell cycle progression [27,28]. As shown in Figure 1A, following Klf4 re-introduction in *Klf4*^*−/−*^MEFs, the growth rate was significantly reduced. Also, consistent with previous reports that KLF4 exerts a cell cycle checkpoint effect by activating the expression of p21 [16,29], the level of p21 was upregulated when compared to control *Klf4*^*−/−*^MEFs (Figure 1C). However, given the modest p21 upregulation in Klf4-transfected *Klf4*^*−/−*^MEFs when compared to control *Klf4*^*+/+*^MEFs (Figure 1C), it is possible that such a robust inhibition of cell proliferation observed in *Klf4*^*−/−*^MEFs following Klf4 over expression might not be attributed to a single factor alone. In response to the high basal DNA damage and genetic instability observed in *Klf4*^*−/−*^MEFs, such a robust suppression of proliferation following Klf4 overexpression could be the result of a cumulative effect of the upregulation of additional cell-cycle progression inhibitors that have been shown to be upregulated by Klf4, such as 14-3-3-sigma [27] and other Cip/Kip family members p57 [27] and p27 [30], this in addition to the suppression of promoters of the cell cycle progression such as cyclin E (Figure 1C and [22]), cyclin B1 [26] and cyclin D1 [31], Such notion requires further investigation.

We have previously shown that *Klf4*^*−/−*^have a higher level of apoptosis than *Klf4*^*+/+*^MEFs [18,23]. Here we demonstrated that overexpression of Klf4 has no apparent effect on basal level of apoptosis in *Klf4*^*+/+*^but trended to reduce apoptosis in *Klf4*^*−/−*^MEFs (Figure 1B). These results suggest that under basal conditions, overexpression of Klf4 in cells with endogenous Klf4has no additional advantage on reduction of apoptosis and that overexpression of Klf4 in absence of endogenous Klf4 might be advantageous to cell survival by reducing the apoptotic level.

Abnormal duplication of centrosomes is a major reason for chromosome instability in cancer because it leads to multipolar spindles which direct unequal segregation of chromosomes during mitosis, thus increasing the frequency of mitotic defects [32,33]. We have previously shown that Klf4 plays a role in regulating centrosome duplication in MEFs as evidenced by the presence of centrosome amplification (defined as ≥3 centrosomes per cell) in *Klf4*^*−/−*^MEFs when compared to *Klf4*^*+/+*^MEFs [18]. Centrosome amplification occurs when its duplication becomes dysregulated. It is known that the p53-p21-cyclin E axis of pathway plays a major role in regulating centrosome duplication. p53 is a tumor suppressor that induces the expression of p21 [34,35], which inhibits the activity of Cdk2/cyclin E [36]. Previous work has shown that disruption of this pathway (loss of p53 or p21, or overexpression of cyclin E) can induce centrosome amplification [37]. Recently, we demonstrated that genetic instability in the absence of Klf4 is likely due to elevated cyclin E and p53 levels, which are normally suppressed by Klf4 [18]. The current study demonstrates that Klf4 may play a role in correcting abnormal centrosome amplification in *Klf4*^*−/−*^MEFs. A potential mechanism by which this is achieved is indicated by the results in which Klf4 overexpression in *Klf4*^−/−^MEFs lowered the levels of p53 and cyclin E and increased that of p21, cumulating in a restoration of the normal centrosome duplication process.

The DNA damage response (DDR) is essential for the maintenance of genetic stability and an unstable genome leads to the accumulation of mutations and cancer development [38,39]. Inefficient DNA double-strand break (DSB) repair can result in chromosomal translocation, deletion and chromosome fusion or loss [40]. DDR signaling involves a large number of proteins that act as sensors, mediators, transducers and effector proteins [41,42]. Recruitment of the DDR protein γ-H2AX, BRCA1, and 53BP1 to DNA DSBs is a key event in the DDR [43-45]. Defective recruitment of repair factors at DNA DSBs, such as delay in foci assembly and disassembly, is associated with defective DDR. *Klf4*^*−/−*^MEFs contain a high level of phosphorylated histone 2AX (*y*-H2AX), a marker for double-strand DNA breaks, and exhibit chromosome aberrations including dicentric chromosomes, double minute chromosomes, and chromatid breaks [18]. The current study shows that a significantly higher fraction of *Klf4*^−/−^MEFs contained γ-H2AX foci as compared to *Klf4*^+/+^MEFs over 24 h in response to γ-irradiation (Figure 3B). In *Klf4*^*−/−*^MEFs we observed no appreciable DNA repair response, showing a persistently elevated percentage of cells with 53BP1 foci 24 h post-irradiation compared to a robust DNA repair response in *Klf4*^+/+^MEFs post irradiation (Figure 3C). These results suggest that Klf4 may be involved in the DNA repair response. This role is further substantiated by the ability of Klf4 to correct DNA damage present in *Klf4*^−/−^MEFs after its reintroduction as demonstrated by lower number of foci of both γ-H2AX and 53BP1 with and without irradiation (Figure 4B and C). Taken together these results suggest that KLF4 plays a role in repairing DNA damage but the exact mechanism by which KLF4 accomplishes this requires further exploration.

Genetic instability, which includes both numerical and structural chromosomal abnormalities, is a hallmark of cancer. We recently reported that *Klf4*^*−/−*^MEFs exhibit aneuploidy [18]. The current study demonstrated a role of Klf4 in preserving genetic integrity by correcting aneuploidy in *Klf4*^*−/−*^MEFs. Re-expression of Klf4 in *Klf4*^*−/−*^ cells resulted in a decrease in the number of cells exhibiting aneuploidy when compared to control *Klf4*^*−/−*^MEFs (Figure 5). The role of KLF4 in maintaining genetic stability is further substantiated by the ability of Klf4 to suppress micronuclei formation in *Klf4*^−/−^ cells (Figure 6) as micronuclei is considered a biomarker of chromosomal damage, genome instability, and eventually of cancer risk [46]. The mechanism by which Klf4 maintain ploidy and chromosome integrity is currently being investigated.

## Conclusion

In summary, our lab previously identified KLF4 as a potential tumor suppressor in colorectal cancer in which KLF4 level is reduced in tumor tissues relative to normal tissues [13]. Moreover, in a large cohort of colon cancer, loss of KLF4 expression is an indicator for poor prognosis including survival [47]. These results are consistent with the observations of the current study showing that KLF4 has an important function in maintaining genetic stability. Importantly results of our study also indicate that re-expression of KLF4 reverts the genetic instability encountered in cells that have lost the KLF4 gene. These results suggest that approaches to increase KLF4 levels may potentially serve as a novel therapeutic option for colorectal cancer.

## Methods

### Cells and cell culture

The wild type (*Klf4*^+/+^) and null (*Klf4*^−/−^) for *Klf4* mouse embryonic fibroblasts (MEFs) were generated as previously described [23]. Studies involving experimental animals have been reviewed and approved by the Stony Brook University Institutional Review Board (Protocol #245765). Cells were Maintained in Dulbecco’s Modified eagle’s Medium (DMEM) supplemented with 10% FBS, 1% penicillin/streptomycin at 37°C in atmosphere containing 5% CO_2_. To overexpress Klf4-GFP and GFP-control in MEFs, cells were transiently transfected with 3 μg plasmid DNA (per well in a 6-well plate) or 0.6 μg plasmid DNA (per well in a 4-well glass slide) using Lipofectamine 2000 reagent (Life Technologies) according to Manufacturer’s instructions. For cell proliferation assay, cells were seeded onto 6-well plates at a density of 10^5^ cells per well in triplicate. Cells were harvested by trypsinization every 24 h for 3 days and counted using Z1 Coulter Particle Counter (Beckman Coulter). For DNA-damage analysis cells were treated or not with γ-irradiation using ^137^Cs -irradiator at 0.75 Gy/min for a total of 2 Gy. Media were refreshed and treated cells were allowed to recover for 1, 4 or 24 h before fixation for immunostaining.

### Plasmids

Expression vector pEGFP-N1 was purchased from Clontech. For generation of Klf4-GFP fusionMKLF4 ORF was excised from pGBKT7-Klf4 vector [48] using *Nco*I and *Eco*RI restriction enzymes. ExcisedMKlf4 ORF was then inserted in frame in pRSET B vector, and the stop codon of theMKlf4 was removed by PCR site directed mutagenesis. TheMKlf4minus stop codon was then excised using *Kpn*I restriction enzyme and inserted in pEGFP-N1.

### Cytogenetic analysis

Cytogenetic analysis by Metaphase spreading of MEFs was performed as described previously [49]. Colcemid (0.5 μg/ml, Life Technologies) was added to MEFs 4 h before harvesting. After treatment, floating rounded-up Mitotic and adherent cells (obtained from the Medium and a PBS wash or after trypsinization, respectively) were pooled and pelleted by centrifugation at 10,000 rpm for 5 min. Cells were swollen in hypotonic solution (0.075 M KCl) at 37°C for 15 min and fixed in fresh, Carnoy’s fixative (methanol: glacial acetic acid at 3:1) for 10 min at room temperature. Cells were spun down at 1000 rpm for 10 min, and washed three times in Carnoy’s fixative and then dropped onto glass slides and aged in a 60°C oven overnight. Cells were subjected to hoechst staining for nucleus visualization. Metaphase spreads images were acquired using a Nikon eclipse 90iMicroscope (Nikon Instruments Inc.) equipped with a DS-Qi1Mc and DS-Fi1, CCD cameras (Nikon Instruments Inc.). The numbers of chromosomes in Metaphase (n = 100 cells) from each genotype were counted and analyzed.

### Immunofluorescence analysis

For all the immunostaining experiments, cells grown on glass coverslips were washed briefly with PBS and fixed with 3.7% formaldehyde for 30 min at room temperature followed by three times wash with PBS. For centrosome count, at 24 h post-transfection, untransfected and transfected cells were fixed and washed as mentioned above. Cells were then incubated with blocking solution (3% bovine serum albumin (BSA), 0.2% Triton X-100 in PBS) for 1 h at room temperature, probed with rabbit anti-γ-tubulin polyclonal antibody (10732; Santa Cruz) overnight at 4°C and detected with Alexa Fluor 568-conjugated goat anti-rabbit IgG antibody (A11011, Life Technologies) for 1 h at 37°C. Cells were then washed once and counterstained with hoechst for 5 min at room temperature in the dark. Finally cells were washed two times and Mounted in Prolong Antifade kit (Life Technologies), and visualized with Nikon microscope. Antibody dilutions and washes after incubations were performed in blocking solution. For γH2AX and 53BP1 foci staining, cells were transfected as mentioned above, and left untreated or γ-irradiated (2 Gy) at 24 h post-transfection, and incubated for 1, 4, or 24 h. Cells were fixed and immunostaining was carried out as Mentioned above. Cells were probed with Mouse anti-γH2AXMonoclonal antibody (05–636; Millipore) or rabbit anti-53BP1 polyclonal antibody (ab21083, Abcam) overnight at 4°C, and detected with Alexa Fluor 568-conjugated goat anti-mouse IgG antibody or Alexa Fluor 568-conjugated goat anti-rabbit IgG antibody, respectively, for 1 h at 37°C. For cleaved caspase 3 staining, cells were transfected as Mentioned above. At 24 h post-transfection, cells were fixed and immunostaining was carried out as Mentioned above. Cells were probed with rabbit anti-cleaved caspase 3 polyclonal antibody (9664S, Cell signaling) overnight at 4°C and detected with Alexa Fluor 568-conjugated goat anti-rabbit IgG antibody for 1 h at 37°C.

### Micronucleus assay

Cells were seeded onto coverslips and transfected as mentioned above. Five hours post transfection cells were treated with 4 μg/ml cytochalasin B (C6762, Sigma) in fresh media and incubated overnight. At 24 h post-cytochalasin B addition, cells were stained with hoechst for 5 min at room temperature in the dark. Finally cells were washed two times mounted in Prolong Antifade kit (Life Technologies) and visualized with Nikon microscope.

### Immunoblotting

Cells were lysed in lyses buffer containing 100 mm Tris–HCl (pH6.8), 2% sodium dodecyl sulfate (SDS) and 20% glycerol, and vortexed for 3–4 min for homogenization. Insoluble material was removed by centrifugation at 12,000 rpm for 5 min, and the supernatant was collected for protein quantification. Following quantification, β-mercaptoethanol and bromophenol blue were added to a final concentration of 5% (v/v) and 0.1% (w/v), respectively, and samples were heated at 95-100°C for 10 min. Samples were cooled to room temperature and then used for SDS-PAGE gel electrophoresis. Following protein transfer, the membranes were immunoblotted with the following primary antibodies: rabbit anti-KLF4 (PM07,MBL), goat anti-p53 (6243, Santa Cruz), Mouse anti-p21 (556431, BD Biosciences), Mouse anti-cyclin E (05–363, Millipore), and rabbit anti-γH2AX (05–636, Millipore), and mouse anti-β-actin (A1978, Sigma-Aldrich). The blots were then incubated with appropriate horseradish peroxidase-conjugated secondary antibodies for 1 h at room temperature. The antibody-antigen complex was visualized by ECL chemiluminescence (Millipore).

### Statistical analysis

Statistical analysis for significance between treatments was performed by *t*-test.

## Competing interests

None of the authors had any competing financial interests in relation to the work describe.

## Authors’ contributions

EE carried out the all the experimental studies, participated in the design of the study and drafted the manuscript. EH participated in cell transfections and in Western blots. AG carried out microscope imaging and the statistical analysis. BY participated in the irradiation experiments. VY conceived of the study, and participated in its design and coordination and drafted the manuscript. All authors read and approved the final manuscript.
